# Acylated Ghrelin Supports the Ovarian Transcriptome and Follicles in the Mouse: Implications for Fertility

**DOI:** 10.3389/fendo.2018.00815

**Published:** 2019-01-15

**Authors:** Luba Sominsky, Jeferson F. Goularte, Zane B. Andrews, Sarah J. Spencer

**Affiliations:** ^1^School of Health and Biomedical Sciences, RMIT University, Melbourne, VIC, Australia; ^2^Monash Biomedicine Discovery Institute and Department of Physiology, Monash University, Melbourne, VIC, Australia

**Keywords:** acyl ghrelin, des-acyl ghrelin, ovarian follicles, reproductive success, RNA-seq

## Abstract

Ghrelin, an orexigenic gut-derived peptide, is gaining increasing attention due to its multifaceted role in a number of physiological functions, including reproduction. Ghrelin exists in circulation primarily as des-acylated and acylated ghrelin. Des-acyl ghrelin, until recently considered to be an inactive form of ghrelin, is now known to have independent physiological functionality. However, the relative contribution of acyl and des-acyl ghrelin to reproductive development and function is currently unknown. Here we used ghrelin-O-acyltransferase (GOAT) knockout (KO) mice that have no measurable levels of endogenous acyl ghrelin and chronically high levels of des-acyl ghrelin, to characterize how the developmental and life-long absence of acyl ghrelin affects ovarian development and reproductive capacity. We combined the assessment of markers of reproductive maturity and the capacity to breed with measures of ovarian morphometry, as well as with ovarian RNA sequencing analysis. Our data show that while GOAT KO mice retain the capacity to breed in young adulthood, there is a diminished number of ovarian follicles (per mm^3^) in the juvenile and adult ovaries, due to a significant reduction in the number of small follicles, particularly the primordial follicles. We also show pronounced specific changes in the ovarian transcriptome in the juvenile GOAT KO ovary, indicative of a potential for premature ovarian development. Collectively, these findings indicate that an absence of acyl ghrelin does not prevent reproductive success but that appropriate levels of acyl and des-acyl ghrelin may be necessary for optimal ovarian maturation.

## Introduction

Since its initial discovery and characterization (1), ghrelin has received increasing attention in metabolic, cardiovascular, stress, motivation, and memory research as having multiple important roles in these fields (2–10). Ghrelin has also been implicated in supporting reproductive function, acting at all levels of the hypothalamic-pituitary-gonadal (HPG) axis (11–14), including both directly and indirectly on the mammalian ovary (11, 15–17). In doing so it mediates changes in the metabolic state and in the levels of stress and reward on puberty, fertility, and fecundity (18).

Ghrelin is a 28 amino acid peptide, produced in the gastrointestinal tract. It is modified by n-octanoylation of serine at the third position (Ser-3) by the enzyme ghrelin-O-acyltransferase (GOAT), to form acylated ghrelin (19, 20). Ghrelin and GOAT are highly conserved in vertebrates (21). In circulation, ghrelin exists in at least two major bioactive forms: acylated and des-acylated ghrelin (22). It is the acylated form that acts at the growth hormone secretagogue receptor (GHSR). The receptor for des-acyl ghrelin has not yet been identified, nevertheless, des-acyl ghrelin has been shown to suppress the effects of acyl ghrelin (23, 24) and to exert independent biological effects on metabolism (25–27), cardiovascular function (9, 28), stress (10), and reproduction (29–31) and is thus an additional important target for investigation.

Both high and low levels of ghrelin appear to be detrimental for fertility, suggesting that a certain balance between circulating acyl and des-acyl ghrelin is important for reproductive potential (18). As such, acute administration of acyl ghrelin in rats impairs folliculogenesis, induces morphometric changes in the ovary, and reduces ovarian volume (32). Chronic administration of acyl or des-acyl ghrelin, or the combination of both, delays follicle maturation and reduces ovarian weight, suggesting the inhibitory effects of ghrelin on the ovary may not be solely dependent on the GHSR-mediated signaling pathway (31). In mice, both administration of a high dose of acyl ghrelin and GHSR antagonism during peri-implantation and early gestation impair fertilization, implantation, and embryo development (33). Human data show that while acyl ghrelin inhibits ovarian steroidogenesis (16), endometrial expression of the ghrelin gene and GHSR1a are decreased in infertile women (34), supporting the hypothesis that an adequate balance within the ghrelin system is required to maintain healthy reproductive function.

The focus on the physiological role of des-acyl ghrelin has only recently begun to gain attention and a large number of studies report the levels and the effects of either total or acyl ghrelin, with only a limited number of studies assessing des-acyl ghrelin (18, 35). Given that both high and low concentrations of ghrelin exert negative effects on fertility, and that some of these effects may be mediated through GHSR-independent pathways, it is imperative to further our understanding of the role des-acyl ghrelin plays in reproduction.

Genetic deletion of GOAT removes the capacity for ghrelin acylation and results in undetectable concentrations of acyl and chronically high levels of des-acyl ghrelin during development and throughout life (36, 37). These GOAT KO mice have been used to investigate the role of the GOAT-ghrelin system in metabolism and stress (36–39), and we used it here to test the hypothesis that acyl ghrelin plays a significant role in the development and function of the reproductive system. GOAT KO mice do not display developmental or overt anatomical differences from WT (40). They do, however, respond differently to conditions of altered metabolic state and stress, as evident from findings in males (10, 37). Thus, we hypothesized that the absence of GOAT (and so the absence of acyl ghrelin and high levels of des-acyl ghrelin) would alter the development of the ovary, leading to detrimental changes in the ovarian transcriptomic profile and impaired development and function of the ovary. We also tested whether these changes were maintained into adulthood and were reflected in differences in the number of ovarian follicles per mm^3^ of ovary or in major reproductive endpoints, including puberty onset and fecundity.

## Materials and Methods

### Animals

In these experiments we used female mice. GOAT KO mice on a C57/Bl6 background were obtained from Regeneron Pharmaceuticals (Tarrytown, NY) and bred (het × het; 1 male × 2 females) in the Monash Animal Services to generate WT and KO littermates, as previously described (10). Mice were group-housed under standard laboratory conditions with *ad libitum* access to food and water at 23°C in a 12 h light/dark cycle. All procedures described here were in accordance with the National Health and Medical Research Council Australia Code of Practice for the Care of Experimental Animals and the Monash University Animal Ethics Committee guidelines.

### Ovarian Tissue Collection

To assess the effects of GOAT deletion on ovarian morphology and transcriptome, we collected ovaries from GOAT KO and WT juvenile (3 weeks old) and adult (10 weeks old) mice. Mice were deeply anesthetized by isoflurane inhalation and ovaries were excised. One ovary from each animal was snap frozen in liquid nitrogen and stored at −80°C for gene analysis, and one ovary was fixed in Bouin's solution (Sigma-Aldrich, St Louis, MO, USA) overnight, rinsed four times in 70% ethanol and stored in ethanol until processing.

### Characterization of Ovarian Morphometry

Exogenous acyl ghrelin suppresses follicle maturation and reduces ovarian volume in the prepubertal ovary (31, 32). It also disrupts granulosa cell steroidogenesis (16). We therefore investigated if the deletion of GOAT and thus a change in acyl and des-acyl ghrelin concentrations would induce morphometric changes in the GOAT KO ovary. We thus assessed the number of ovarian follicles in juvenile and adult mice. As previously described (41, 42), fixed ovaries were dehydrated, embedded in paraffin and sectioned at 4 μm. For morphometric analysis, 20 sections on 10 slides, 36 μm apart, were stained with haematoxylin-eosin (H&E). Two sections per slide were assessed on the basis of an 8 μm distance between the sections, allowing a complete assessment of primordial follicle counts at this location, as per our previous publications (42, 43). Follicles were classified as: (a) *primordial*: an oocyte surrounded by a single layer of flattened pregranulosa cells; (b) *early primary*: an oocyte surrounded by a single layer of flattened pregranulosa cells with at least two cuboidal granulosa cells; (c) *primary*: an oocyte surrounded by cuboidal granulosa cells; (d) *preantral*: follicles with no antral cavity and two or more layers of cuboidal granulosa cells; (e) *antral*: an antral cavity visible, with at least two layers of cuboidal granulosa cells. We scanned whole ovarian sections using an Olympus VS120 slide scanner (Olympus, Tokyo, Japan). Only follicles with visible nuclei and nucleoli were counted to prevent counting the same follicle more than once. Area measurements were obtained using ImageJ (National Institutes of Health, MD, USA). Follicle counts were adjusted per total section volume, calculated as area multiplied by section thickness, according to Bernal et al. (44), Aiken et al. (45, 46), Tsoulis et al. (47), Asadi-Azarbaijani et al. (48), and Chan et al. (49), and presented as counts per mm^3^ (*n* = 4–6 animals per group). Follicles were classified as atretic if they presented with one or more of the following criteria: oocyte degeneration; granulosa cell degeneration, disorganization and retraction from the oocyte; appearance of pyknosis in more than 10% of granulosa cells (49–51).

### Immunohistochemistry

We used proliferating cell nuclear antigen (PCNA) immunolabeling to visualize follicle activation and growth, as previously described (42, 52). We de-waxed paraffin-embedded sections (4 μm) in histolene and rehydrated them in ethanol washes. Antigen retrieval was carried out by microwaving sections in sodium citrate buffer for 15 min (10 mM sodium citrate, pH = 6). Slides were then cooled down to room temperature (RT) and blocked in 3% bovine serum albumin (BSA)/0.03% Triton X-100/ phosphate-buffered saline (PBS) for 1 h at RT. Sections were then incubated overnight at 4°C with mouse monoclonal anti-PCNA (1:200; #ab29, lot #GR201287, Abcam, Cambridge, UK). We then washed the slides in PBS/0.1% Triton X-100 and incubated them with Alexa Fluor 594 donkey anti-mouse IgG fluorescent-conjugated secondary antibody (1:200; A21203 Thermo Scientific, Rockford, IL, USA). Sections were then counterstained with 4′,6-diamidino-2-phenylindole (DAPI) using Fluoroshield with DAPI mounting medium (Sigma-Aldrich, St Louis, MO, USA) and viewed under an Olympus BX61 fluorescent microscope fitted with a Nikon DS-Ri2 camera. A minimum of two randomly selected slides were evaluated for each animal. We assessed fluorescence intensity in PCNA-positive follicles with a visible nucleus using ImageJ (National Institutes of Health, MD, USA). Mean fluorescence intensity was calculated using a corrected total cell fluorescence (CTCF) formula (CTCF = integrated density—(area of selected section × mean fluorescence of background readings), as previously described (53). Data were normalized to the mean fluorescence intensity of the control group and expressed in arbitrary units (AU) (43, 54, 55).

For detection of follicular apoptosis, we de-paraffinised and rehydrated paraffin-embedded sections as described above. We pre-treated the sections with 20 μg/mL proteinase K for 15 min at RT and performed analysis for terminal deoxynucleotidyl transferase dUTP nick end labeling (TUNEL) using ApopTag Fluorescein *In situ* Apoptosis Detection Kit (Merck Millipore, Burlington, MA, USA) according to the manufacturer's instructions. We then counterstained the sections using Fluoroshield with DAPI mounting medium (Sigma-Aldrich). Positive controls consisted of sections treated with DNAse I (Qiagen, Carlsbad, CA, USA) to induce non-specific DNA fragmentation, and negative control staining was performed without active Terminal deoxynucleotidyl Transferase (TdT) but including proteinase K digestion, to control for non-specific incorporation of nucleotides. Slides were viewed under a fluorescent microscope and follicles were classified as apoptotic if they contained a TUNEL-positive oocyte and/or ≥4 TUNEL-positive granulosa cells for primary, secondary and antral follicles, or >1 TUNEL-positive granulosa cells for primordial follicles (42, 56, 57).

### RNA Isolation

We isolated total RNA using QIAzol reagent and RNeasy Mini Kits (QIAGEN, Valencia, CA, USA). RNA concentrations were determined using a spectrophotometer, NanoDrop 2000/2000c (Thermo Fisher Scientific, Wilmington, DE USA) and 1 μg RNA was transcribed to cDNA using an iScript cDNA synthesis kit (Bio-Rad Laboratories, Hercules, CA, USA), according to the manufacturer's instructions.

### Next-Generation Sequencing

Total RNA (1 μg) samples isolated from juvenile GOAT KO and WT mouse ovaries (*n* = 4 per group) were submitted to the Australian Gene Research Facility (AGRF; Melbourne, VIC, Australia) for processing and bioinformatics analysis. The quality of the total RNA was assessed by a Bioanalyser (RNA Integrity Number = 10 for all samples). The samples were then sequenced on an Illumina HiSeq 2000 platform (Illumina, San Diego, CA) generating 50 bp single-reads per lane. The primary bioinformatics analysis involved de-multiplexing and quality control. The per base sequence quality indicated that the Phred quality score was above 30 for >96% of bases across all samples (58, 59). The reads were also screened for the presence of any Illumina adaptor/overrepresented sequences and cross-species contamination. The cleaned sequence reads were then aligned against the *Mus musculus* genome (Build version mm10). The Tophat aligner (v2.0.14) was used to map reads to the genomic sequences. The counts of reads mapping to each known gene were summarized. The transcripts were assembled with the Stringtie tool v1.0.4 (http://ccb.jhu.edu/software/stringtie/) utilizing the reads alignment with reference annotation based assembly option (RABT). The GENCODE annotation containing both coding and non-coding annotations for mouse genome version GRcm38 (Ensemble release 81) was used as a guide.

To estimate differences in gene counts between groups, differential expression analysis was undertaken using specialized R libraries from Bioconductor version 3.2 (http://www.bioconductor.org) (60). A multidimensional scaling plot revealed that two samples (one from each of the GOAT KO and WT groups) did not cluster with the rest of the samples from that group and were thus considered as outliers for further analysis. The data filter was set to 0.5 < logFC < −0.5 difference and *p* < 0.05. A test for over-representation of Gene Ontology (GO) terms was performed using the GOANA method (https://www.bioconductor.org/packages/devel/bioc/manuals/limma/man/limma.pdf). The clusterProfiler software package was used to analyse and visualize functional profiles (GO and Kyoto Encyclopedia of Genes and Genomes, KEGG) of gene and gene clusters (http://www.bioconductor.org/packages/release/bioc/html/clusterProfiler.html). The clusterProfiler supports enrichment analysis of Reactome and KEGG with either hypergeometric test or Gene Set Enrichment Analysis (GSEA) (61).

In addition to these analyses, we also used the Ingenuity Pathway Analysis (IPA; Qiagen Inc., https://www.qiagenbioinformatics.com/products/ingenuity-pathway-analysis) platform to explore further downstream and upstream effects of GOAT deletion in our dataset. The recommended set size for IPA core analysis from gene expression data is 200–3,000 molecules, and different fold change cut-offs are routinely used to allow inclusion of more differentially expressed genes for meaningful pathway analyses [e.g., (62, 63)]. We therefore performed the core analysis at 0.3 < logFC < −0.3 difference and *p* < 0.05, resulting in 656 analysis-ready molecules. These cut-off criteria allowed us to predict directionality of change in downstream functions and upstream regulators, accounting for a potential dilution of information as a result of whole ovary sequencing. Statistical significance was calculated using the right-tailed Fisher's Exact Test. The activation z-score, a statistical measure that assesses the match between observed and predicted upstream or downstream regulation patterns based on previous literature was also used to evaluate significance of effects on diseases and biological functions, as well as the activation and inhibition states of predicted upstream regulators [see (64) for further information on the IPA core analysis]. The data discussed in this publication have been deposited in NCBI's Gene Expression Omnibus and are accessible through GEO Series accession number GSE106339.

### Real-Time Quantitative PCR Array

We also used a Custom RT^2^ PCR array (CLAM26350; Qiagen) designed to specifically examine, in juvenile and adult ovaries, the changes in the top ten over- and under-expressed genes (Table 1) that were identified by RNA sequencing in the ovaries of juvenile GOAT KO mice. Pseudogenes were excluded from the analysis. We also used this ovary to confirm the absence of GOAT (*Mboat4*) in the ovaries of GOAT KO mice. Total RNA, 400 ng, extracted as detailed above, was transcribed to cDNA using the RT^2^ First Strand Kit (Qiagen), according to the manufacturer's instructions. Samples were then diluted as per manufacturer's instructions in RT^2^ SYBR Green Mastermix, loaded onto 384-well PCR array plates and amplified on the QuantStudio™ 7 Flex Real-Time PCR System (Applied Biosystems, Carlsbad, CA, USA), including an initial activation step at 95°C for 10 min followed by 40 cycles at 95°C for 15 s and 60°C for 1 min. *Actb* and *Gapdh* were used as endogenous controls. The *C(t)* values for these genes were averaged and used for the comparative threshold cycle (ΔΔ *C(t)*) calculations, where *C(t)* is ≤ 40. Fold changes were then calculated using the 2^−ΔΔ*C(t)*^ Equation (98).

**Table 1:** Top 10 over- and under-expressed genes in GOAT KO mouse ovary (*p* < 0.05) and supporting literature.

**Gene symbol**	**Gene name NCBI**	**Log_**2**_FC**	**Summary of function**
*Gm10036*	Predicted gene 10036	2.75	Ribosomal protein L11 pseudogene (65).
*Grik3*	Glutamate receptor, ionotropic, kainate 3	1.74	Also known as *Glur7*. Plays a role in neuroactive ligand-receptor interaction, underlying glutamate-mediated excitatory synaptic transmission, and is expressed in the ovary (66, 67)
*Spocd1*	SPOC domain containing 1	1.72	Involved in negative regulation of phosphatase activity. SPOC domain (Spen paralogue and ortholog C terminal) plays a role in developmental signaling (68, 69).
*Grem1*	Gremlin 1, DAN family BMP antagonist	1.60	Expressed in granulosa cells. Regulates folliculogenesis and primordial to primary follicle transition (70, 71)
*Cyp19a1*	Cytochrome P450, family 19, subfamily a, polypeptide 1	1.40	Encodes aromatase, the key enzyme in estrogen biosynthesis. Significantly increased during preovulatory follicle development (72–74).
*Inhba*	Inhibin, beta A	1.37	Encodes activin β A subunit that negatively regulates pituitary follicle-stimulating hormone (FSH) synthesis. Prominently expressed in granulosa cells of preantral and antral follicles. Deletion causes neonatal lethality, significant craniofacial defects and abnormal folliculogenesis (75–77).
*Hspb7*	Heat shock protein family B (small) member 7	1.29	Potent suppressor of protein aggregation, assists in the clearance of stress-induced misfolded proteins (78, 79).
*Gm15421*	Predicted gene 15421	1.17	Ribosomal protein L22 like 1 pseudogene (80).
*Sohlh1*	Spermatogenesis and oogenesis specific basic helix-loop-helix 1	1.08	Expression is confined to primordial oocytes and is required for their differentiation. In adult ovary transcript expression is decreased as the oocytes are recruited to form primary and secondary follicles (81, 82).
*Drd4*	Dopamine receptor D4	1.05	Androgen-dependent gene (83–85). Strongly expressed in mouse adult ovary, with no known function (67).
*Leprotl1*	Leptin receptor overlapping transcript-like 1	−0.98	Negatively regulates growth hormone (GH) receptor expression and is overexpressed during fasting (86). Transgenic mice overexpressing leprotl1 show growth retardation (87). Expressed in granulosa cells throughout follicular development from small to ovulatory follicles and significantly increased in corpus luteum, compared to small follicles (88).
*Dcdc2b*	Doublecortin domain containing 2b	−1.02	Encodes a member of the doublecortin family. The doublecortin domain binds tubulin and increases microtubule polymerisation (89).
*Gm5620*	Predicted gene 5620	−1.06	tubulin, alpha 1B pseudogene. Non-protein coding.
*Cited4*	Cbp/p300-interacting transactivator, with Glu/Asp-rich carboxy-terminal domain, 4	−1.07	Luteinising hormone (LH) target gene during ovulation. The pre-ovulatory LH surge induces *Cited4* expression in cumulus and granulosa cells and this expression is required for cumulus-oocyte complex expansion and ovulation. Regulates histone acetylation in the promoters of the LH-induced target genes as a histone acetyltransferase in mouse granulosa cells undergoing luteinization after the ovulatory LH surge (90).
*Gm13152*	Zinc finger protein 982	−1.09	Expressed in mouse fetal ovary in meiotic prophase, with increasing expression between E12.5 to E16.5 (91). No specific function in adult ovary has been attributed.
*Gm13103*	Predicted gene 13103	−1.11	Highly expressed in adult mouse ovary. Specifically expressed in fully grown oocytes (92)
*Rab3b*	RAB3B, member RAS oncogene family	−1.15	A marker for regulated secretion, expressed in cells with a high activity of regulated exocytosis. In the pituitary, Rab3b is essential for GnRH-regulated exocytosis in gonadotrophs (93). In the sheep ovary, Rab3b has been co-localized with oxytocin to the same luteal staining granules of the corpus luteum during the luteal phase of the estrous cycle (94)
*Rab6b*	RAB6B, member RAS oncogene family	−1.53	Controls retrograde transport from the Golgi body to the endoplasmic reticulum and is predominantly expressed in neuronal cells (95, 96).
*Gm6166*	Predicted gene 6166	−1.81	Fatty acid-binding protein, epidermal-like. Non-protein coding.
*Myh6*	Myosin, heavy polypeptide 6, cardiac muscle, alpha	−2.05	Involved in protein dimerization activity. Overexpressed in the ovaries of 5α-dihydrotestosterone treated rats, mimicking the hyperandrogenic state in women with polycystic ovarian syndrome (97).

### Onset of Puberty and Breeding Capacity

Changes in the availability of acyl ghrelin have been implicated in the timing of puberty onset (99, 100). We therefore examined if the deletion of GOAT and thus an absence of acyl ghrelin affected the onset of puberty in our study. Mice (*n* = 7–8 per group) were inspected daily, beginning at postnatal day (P)25, to determine the day of vaginal opening (a physical marker of puberty onset). When vaginal opening occurred, mice were weighed and left undisturbed until adulthood. It has been previously reported that GOAT KO mice are capable of breeding normally (37), but we wanted to test if this capacity was affected in our housing facility. We therefore also evaluated historic breeding records from Monash Animal Research Platform of 15 WT and 16 GOAT KO females ranging between 2.5 and 6 months of age at first mating, that were continuously mated for 3–5 months with WT and GOAT KO male studs, respectively. Breeding success was indicated by the mean number of pups per litter per dam, as well as by the mean number of pups in the first litter, since C57/Bl6 dams have been shown to have a higher pup mortality rate in their first than in subsequent litters (101).

### Statistical Analysis

In addition to the analysis of RNA sequencing data described above, we used Student's unpaired *t*-tests for the assessment of ovarian morphometry, RT^2^ PCR array, puberty onset, and breeding data between GOAT KO and WT mice. We also used Pearson's correlation analysis to assess the relationship between RT^2^ PCR array and RNA sequencing fold changes. Data are presented as the mean ± SEM. Statistical significance was assumed when *p* ≤ 0.05.

## Results

### Juvenile GOAT KO Mice Have a Reduced Number of Ovarian Follicles (Per mm^3^)

**Figure 1:**
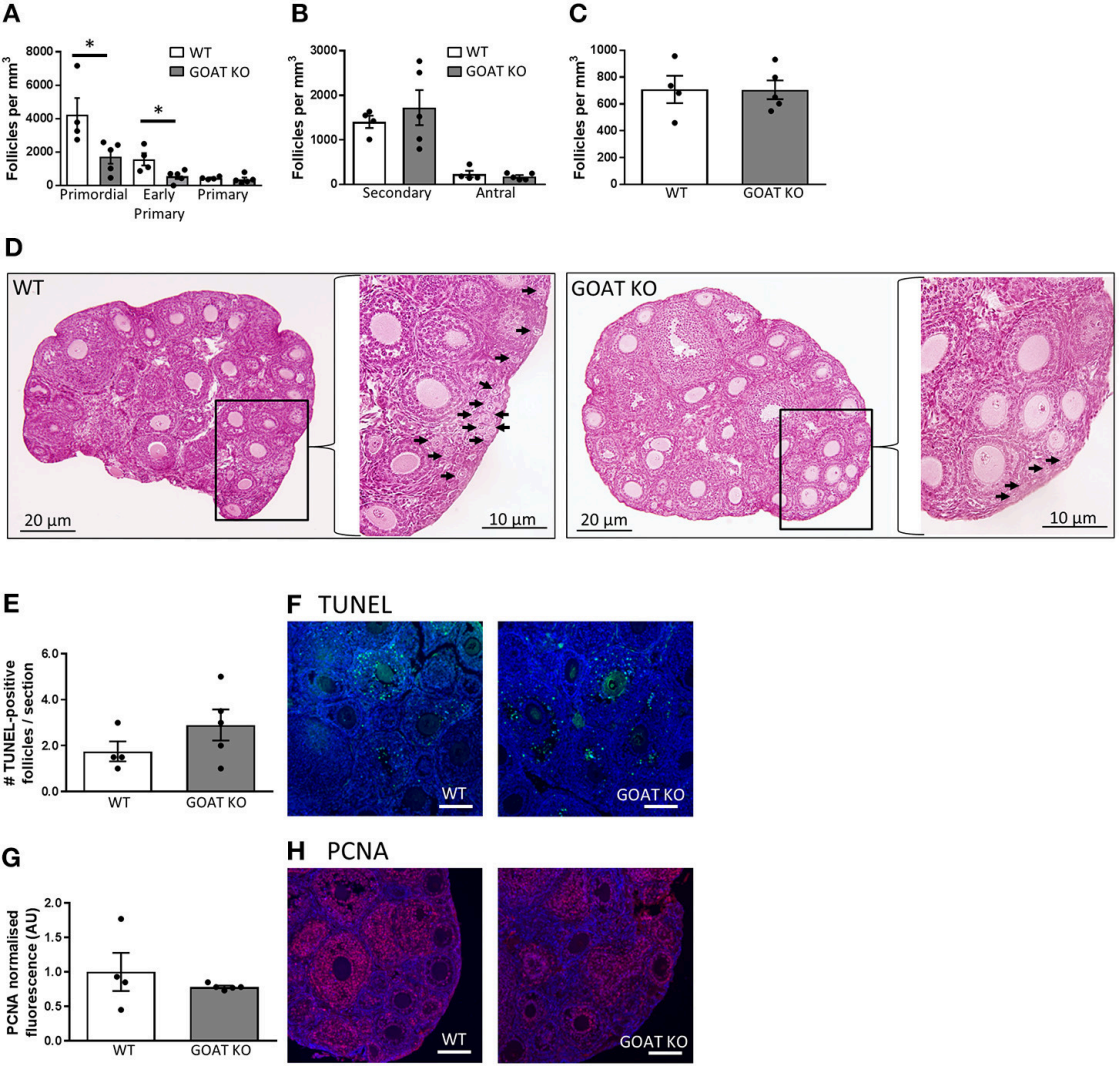
Effects of GOAT deletion on ovarian follicles in juvenile mice. **(A)** Small follicle; **(B)** Large follicle; and **(C)** Atretic follicle counts in the ovary of juvenile GOAT KO and WT mice. Follicle numbers are expressed per mm^3^ of ovarian tissue. Panel **(D)** shows morphological representation of H and E stained juvenile WT and GOAT KO ovary. Black arrows in inserts at higher magnification indicate small follicles (i.e., primordial, early primary and primary) in ovarian cortical region. Scale bars = 20 μm in low and 10 μm in high magnification images. **(E)** Number of TUNEL-positive follicles in the ovary of juvenile GOAT KO and WT mice. **(F)** Representative images of TUNEL-positive staining (green) in the juvenile ovary. **(G)** Normalized mean fluorescence intensity of proliferating cell nuclear antigen (PCNA) staining in juvenile GOAT KO and WT ovaries. **(H)** Representative images of PCNA staining (red) in the juvenile ovary. Scale bars = 50 μm. Data are expressed as mean ± SEM. *p < 0.05.

Body weights in the juvenile phase were not affected by the absence of GOAT (data not shown). However, the juvenile GOAT KO mice had a significant reduction in their small follicle numbers per mm^3^ of ovarian tissue, such that GOAT KO mice had a reduction of more than 50% in the number of primordial [*t*_(_7) = 2.52, *p* = 0.039, Figures 1A,D] and early primary follicles [*t*_(_7) = 2.72, *p* = 0.029, Figures 1A,D; expressed per mm^3^], compared to age-matched WT controls. There were no differences in the numbers of large healthy follicles or atretic follicles (Figures 1B,C; expressed per mm^3^).

Consistent with a lack of differences in large healthy and atretic follicles, there were no significant differences in the number of TUNEL-positive follicles between WT and GOAT KO mice, in the juvenile phase (Figures 1E,F). TUNEL-positive granulosa cells were primarily expressed in large follicles (i.e., secondary and antral), consistent with previous studies showing that the current commonly used apoptotic markers are unable to detect primordial and primary follicle atresia and therefore follicle counts provide the most accurate assessment of primordial and primary follicle loss (102, 103). PCNA, a marker of follicle growth, was also highly expressed in the granulosa cells and oocytes of large follicles and some primary follicles, as previously described (104), with no differences in PCNA expression between juvenile WT and GOAT KO mice ovaries (Figures 1G,H).

### Juvenile GOAT KOs Have Differences in the Ovarian Transcriptome Compared to WT Mouse Ovaries

**Figure 2:**
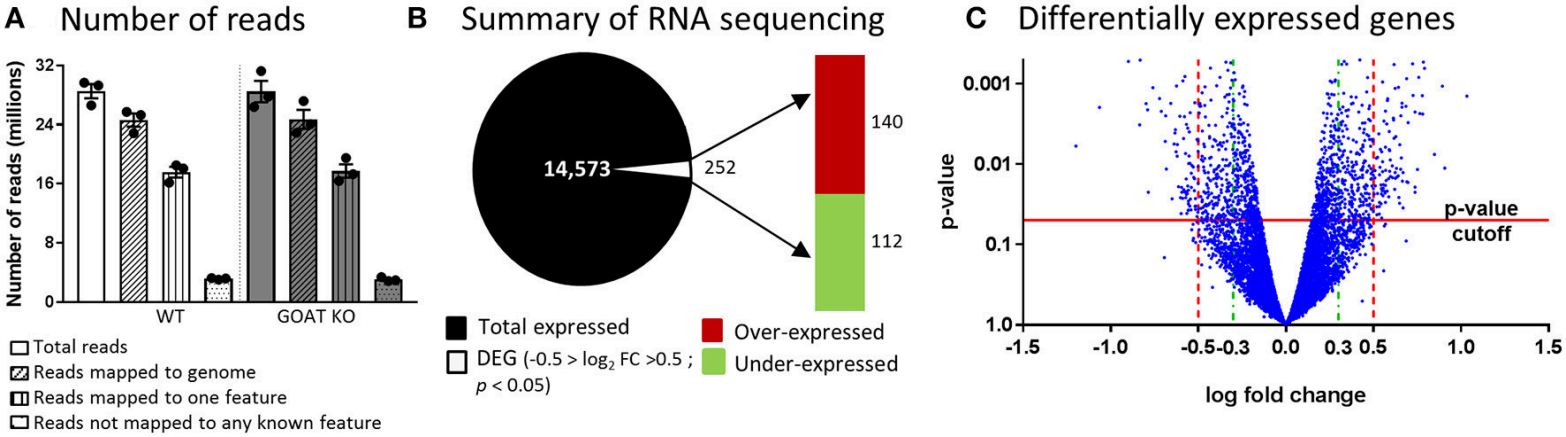
RNA sequencing analysis of ovaries from juvenile GOAT KO versus WT mice. **(A)** Summary of number of reads and their mapping to genome. **(B)** Summary of RNA sequencing. Total number of identified transcripts are presented as black and differentially expressed genes (DEGs; −0.5 > logFC > 0.5; *p* < 0.05) are presented as white. The red bar represents the number of over-expressed transcripts and the green bar represents the number of under-expressed transcripts in the ovaries of juvenile GOAT KO mice. **(C)** Volcano plot for differentially expressed genes, where the *p*-value (Y axis) is plotted against the log fold change (X axis). *p*-value cutoff of 0.05 is represented by horizontal red line. Log fold change filters are represented by vertical dashed lines. Red dashed lines represent a filter set to ±0.5 that was used to test for over-representation of Gene Ontology terms and for pathway enrichment analysis. Green dashed lines represent a filter set to ±0.3, for the analysis using the Ingenuity Pathway Analysis (IPA) platform.

Since reliable markers of apoptosis and proliferation in the small follicle population remain to be developed, we assessed if the reduction in the follicle numbers was associated with changes in ovarian genes and pathways related to reproduction. We thus performed RNA sequencing, characterizing the ovarian transcriptome of juvenile GOAT KO compared to WT mice. The counts of reads mapped to each known gene are summarized (Figure 2A). Differential expression analysis for estimating differences in transcripts across groups identified 14,573 genes, with a significant difference in the expression of 252 genes of at least 1-fold change (−0.5 < logFC < 0.5), *p* < 0.05. A summary of the RNA sequencing results and the distribution of differentially expressed genes (DEGs) are presented in Figures 2B,C. The top ten over- and under-expressed genes in the ovaries of GOAT KO mice are presented in Table 1 along with a summary of their known functions. These DEGs included several genes associated with major biological processes and functions regulating reproductive development. For instance, *Grem1, Cyp19a, Inhba*, and *Sohlh1* play a critical role in folliculogenesis. *Grem1*, regulates primordial to primary follicle transition. *Sohlh1*, is required for oogenesis and is essential for primordial follicle activation. *Inhba* expression is associated with follicular growth, regulating cell proliferation, and follicle stimulating hormone (FSH) action in the ovary. *Cyp19a1*, encodes aromatase cytochrome P450, catalyzing a critical step in ovarian estrogen biosynthesis (71, 72, 81, 105).

**Figure 3:**
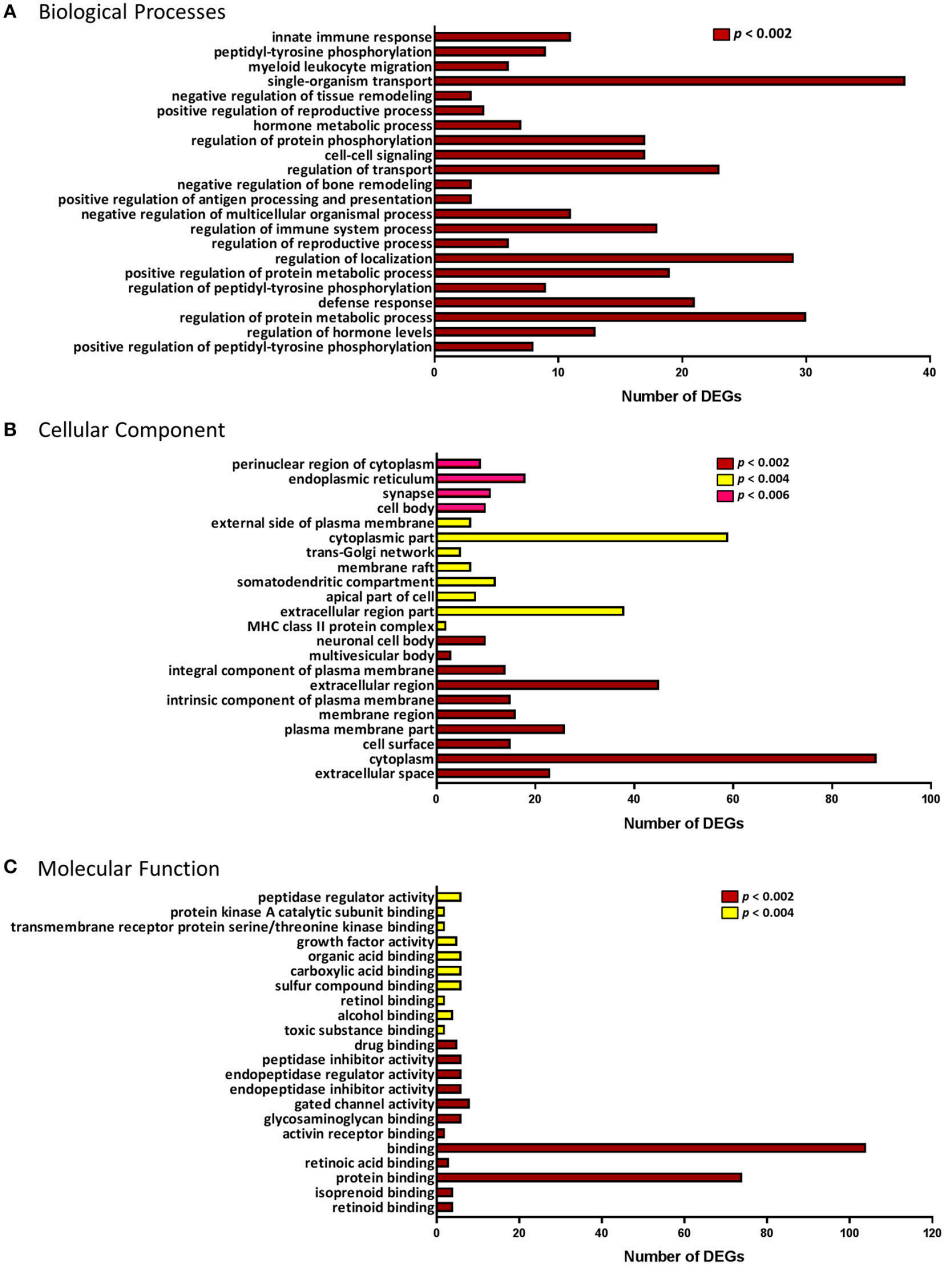
Gene Ontology (GO) enrichment analysis of the differentially expressed genes. GO terminology and the number of differentially expressed genes (DEGs) associated with each of the three categories: **(A)** Biological processes. **(B)** Cellular Component. **(C)** Molecular Function. *p*-value for over-representation of GO terms in DEGs is represented by: red, *p* < 0.002; yellow, *p* < 0.004; and pink, *p* < 0.006.

DEGs were annotated by association with three GO term categories: biological process, molecular function and cellular component. The top 22 GO terms for each category are presented in Figure 3. These included biological processes regulating reproduction, particularly its positive regulation; immune response (e.g., *defense response, regulation of immune system process, positive regulation of antigen processing and presentation, myeloid leukocyte migration, innate immune response*); cell signaling and transport, and others (Figure 3A).

In the ontology of cellular component, GO categories of *extracellular space, plasma membrane, trans-Golgi network*, and *major histocompatibility complex (MHC) class II protein complex*, were among the most significant overrepresented GO terms in the DEGs (Figure 3B). Molecular functions, such as *retinoid binding, activin receptor binding, endopeptidase/peptidase regulator and inhibitor activity* and *growth factor activity* were among the top 22 most significant GO terms (Figure 3C). Using enrichment analysis for Reactome and KEGG pathways we identified several pathways involved in the immune response and cell signaling. Pathway enrichment results are summarized in Table 2.

**Table 2:** Pathway enrichment analysis according to Reactome and Kyoto Encyclopedia of genes and Genomes (KEGG).

**Pathway**	**ID**	**Statistics**	**Annotated genes**
**(A) ENRICHED REACTOME PATHWAYS**			
Neuronal System	5604671	GeneRatio = 6/55; BgRatio = 237/6,598; *p*-value = 0.013; p.adjust = 0.121	*Kcnab3, Kcnn3, Camkk1, Gls2, Gabrb1, Kcnmb2*
Immune System	5604803	GeneRatio = 14/55; BgRatio = 1007/6,598; *p*-value = 0.033; p.adjust = 0.150	*Prkar2b, C3, Sh3gl2, Fbxo44, Kif5a, Ctss/H2-Ab1, Peli3, C8g, Trem2, Il6ra, Tlr7, Itgb2, Cd74*
Adaptive Immune System	5604808	GeneRatio = 8/55; BgRatio = 552/6,598; *p*-value = 0.085; p.adjust = 0.193	*C3, Sh3gl2, Fbxo44, Kif5a, Ctss, H2-Ab1, Itgb2, Cd74*
Hemostasis	5605036	GeneRatio = 7/55; BgRatio = 460/6,598; *p*-value = 0.086; p.adjust = 0.193	*Prkar2b, Igf2, Kif5a, H3f3a, Serpinc1, Itgb2, Kcnmb2*
Innate immune system	5604802	GeneRatio = 7/55; BgRatio = 487/6,598; *p*-value = 0.108; p.adjust = 0.194	*Prkar2b, C3, Ctss, C8g, Trem2, Tlr7, Itgb2*
**(B) ENRICHED KEGG PATHWAYS**			
Drug metabolism—cytochrome P450	mmu00982	GeneRatio = 4/58; BgRatio = 29/3,997; *p*-value < 0.001; p.adjust < 0.05	*Gsta2, Gsta4, Fmo1, Adh1*
Retinol metabolism	mmu00830	GeneRatio = 3/58; BgRatio = 15/3,997; *p*-value < 0.01; p.adjust < 0.05	*Adh1, Lrat, Cyp26b1*
Complement and coagulation cascades	mmu04610	GeneRatio = 4/58; BgRatio = 35/3,997; *p*-value < 0.01; p.adjust < 0.05	*Serpina5, C3, C8g, Serpinc1*
Systemic lupus erythematosus	mmu05322	GeneRatio = 4/58; BgRatio = 37/3,997; *p*-value < 0.01; p.adjust < 0.05	*C3, H2-Ab1, H3f3a, C8g*
*Staphylococcus aureus* infection	mmu05150	GeneRatio = 3/58; BgRatio = 21/3,997; *p*-value < 0.01; p.adjust < 0.05	*C3, H2-Ab1, Itgb2*
Metabolism of xenobiotics by cytochrome P450	mmu00980	GeneRatio = 3/58; BgRatio = 25/3,997; *p*-value < 0.01; p.adjust < 0.05	*Gsta2, Gsta4, Adh1*

GeneRatio: ratio between the number of DEGs in the pathway and the number of DEGs. BgRatio: ratio between the number of genes in the pathway and the total examined background of genes. p.adjust: p-value for hypergeometric test adjusted for Benjamini–Hochberg correction.

### Ingenuity Pathway Analysis of DEGs From Juvenile GOAT KO and WT Mice

**Figure 4:**
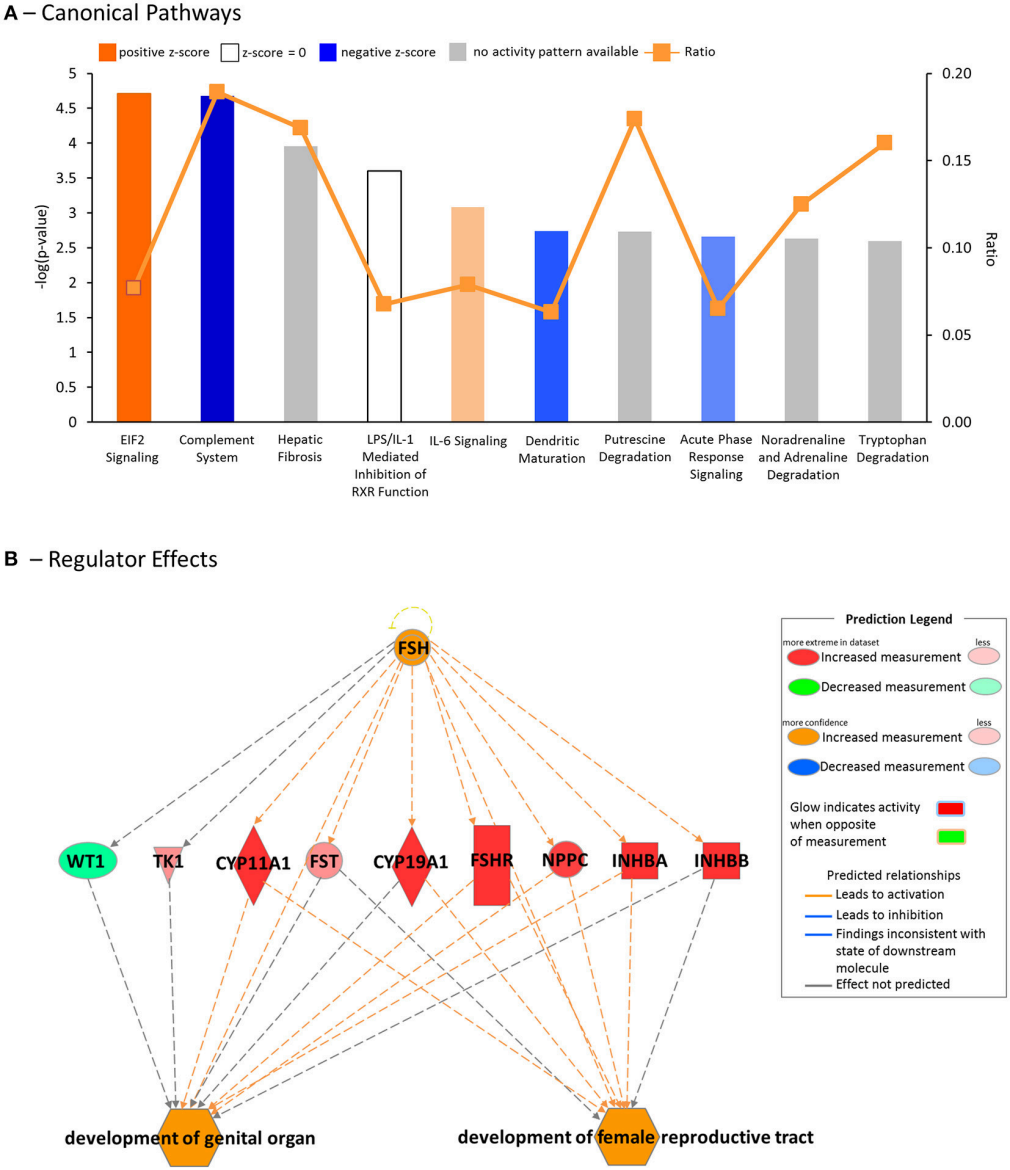
Downstream effects and upstream regulators, as predicted by the Ingenuity Pathway Analysis (IPA) platform. **(A)** Top canonical pathways affected by GOAT deletion. **(B)** Regulator Effects analysis, featuring the top predicted upstream regulator, Follicle Stimulating Hormone (FSH), its connection to the differentially expressed genes (DEGs) in the dataset and their influence on the top biological functions, likely to be affected by GOAT deletion.

Using IPA, we found several canonical pathways were affected by GOAT deletion. The most common of these pathways were those involved in the immune response [*Complement System*, interleukin (*IL*)*-6 Signaling, Dendritic Cell Maturation, Acute Phase Response Signaling*; Figure 4, Supplementary Table 1], similar to the results of the pathway enrichment analysis for Reactome and KEGG pathways, as described above.

DEGs between the GOAT KO and WT juveniles were found to be mostly related to diseases and disorders associated with inflammation (*Inflammatory Disease*, Fisher's Exact Test *p*-value range = 1.28E-03 to 1.81E-10, 112 molecules; *Inflammatory Response, p*-value range 1.20E-03 to 1.37E-09, 133 molecules) and organismal injury (*Organismal Injury and Abnormalities, p*-value range 1.32E-03 to 1.81E-10, 456 molecules; *Connective Tissue Disorders, p*-value range 1.00E-03 to 1.81E-10, 111 molecules), as well as to contribute to *Organismal Development* (*p*-value range 1.30E-03 to 2.90E-07, 166 molecules). This latter biological process included 26 functions associated with *Reproductive System Development and Function*. The functions in this category with an absolute z-score of >1 are presented in Table 3.

**Table 3:** Top functions associated with Reproductive System Development and Function, identified by Ingenuity Pathway Analysis platform.

**Diseases or function annotation**	***p*-value**	**Activation z-score**	**Genes and their direction of change**	**# Genes**
Development of genital organ	0.001	2.226	↓*Bmp7*, ↓*C14orf39*, ↑*Cyp11a1, ↑Cyp19a1, ↓Cyp26b1, ↑Dnah9, ↓Dnajc19, ↑Dnd1, ↑Fads2, ↑Fkbp6, ↑Fshr, ↑Fst, ↓H3f3a, ↑Inhba, ↑Inhbb, ↑Mael, ↑Mcmdc2, ↑Mov10l1, ↓Ngf, ↑Nppc, ↑Piwil2, ↓Serpina5, ↑Smc1b, ↑Sohlh1, ↓Sox9, ↓Stra6, ↑Tarbp2, ↑Tbpl2, ↑Tk1, ↑Ubb, ↓Wt1, ↓Zmynd15*	32
Development of female reproductive tract	0.001	2.113	*↑Cyp11a1, ↑Cyp19A1, ↑Fads2, ↑Fshr, ↑Fst, ↑Inhba, ↑Inhbb, ↓Ngf, ↑Nppc, ↑Sohlh1, ↓Stra6, ↑Tbpl2, ↑Ubb*	13
Quantity of antral follicle	< 0.001	1.342	*↓Bmp7, ↑Cyp19A1, ↑Inhba, ↓Ngf, ↓Ngfr*	5
Fertility	< 0.001	1.280	*↑Ctfr, ↓Chdh, ↑Cyp19a1, ↓Dio3, ↑Dppa3, ↑Fads2, ↑Fshr, ↓H3f3a, ↓H2-Ab1, ↑Hsd17b1, ↑Inhba, ↑Inhbb, ↑Khdc3l, ↓Kmt2c, ↑Pip5k1b, ↓Rarg, ↑Scara5, ↓Serpina5, ↑Spink13, ↑Srd5a1, ↑Stc1, ↑ Tk1, ↓Wt1*	23
Quantity of gonad	< 0.001	1.173	*↓Bmp7, ↑Cdkn2d, ↑Cftr, ↑Cyp19a1, ↓Cyp26b1, ↑Fads2, ↑Fshr, ↑Hsd17b1, ↑Igf2, ↓Il6r, ↑Inhba, ↑Inhbb, ↑Itpa, ↓Ngf, ↓Wt1*	16
Quantity of ovarian follicle	< 0.001	1.122	*↓Bmp7, ↑Cftr, ↑Cyp19a1, ↑Fads2, ↑Fshr, ↑Hsd17b1, ↑Inhba, ↑Inhbb, ↓Ngf, ↓Ngfr*	10
Estrous cycle	0.001	1	*↑C3, ↑Cftr, ↑Cyp19a1, ↑Fads2, ↑Fshr, ↑Inhba, ↓Ngf, ↓Ngfr*	8
Quantity of corpus luteum	< 0.001	0.896	*↑Cftr, ↑Cyp19a1, ↑Fshr, ↑Hsd17b1, ↑Inhba, ↑Inhbb*	6
Quantity of primary ovarian follicle	< 0.001	−1	*↓Bmp7, ↑Cyp19a1, ↑Fshr, ↓Ngf*	4

*The activation z-score makes predictions by using information about the direction of gene regulation in the dataset. Positive z-score predicts an increase in the biological process or disease, while a negative z-score predicts a decrease (inhibition). −2 ≥ z-score ≥ 2 indicates a significant change. A red up arrow indicates an increase in the dataset; A green down arrow indicates a decrease in the dataset. p < 0.05 indicate a statistically significant, non-random association between a set of genes in the dataset and a related function*.

To gain further insight into the biological impact of DEGs in the dataset, we performed a Regulator Effects analysis. The Regulator Effects algorithm connects potential upstream regulators with DEGs in and downstream functions that are affected in the dataset. This algorithm thus aims to provide a hypothesis that may explain how an upstream regulator affects the downstream gene expression and the impact of this activation or inhibition on biological functions and diseases (64). Upstream regulators were limited to genes, RNAs and proteins, while the diseases and functions category was limited to include the previously identified diseases and disorders associated with inflammation and organismal injury and development (Table 3). A cut-off setting of *p* < 0.05 and an absolute z-score of > 2 were applied. The analysis identified $10 potential regulators. FSH was identified as the main potential regulator of several genes that are likely to be involved in the *development of female reproductive tract* and *development of genital organ*, the top biological functions that are likely to be affected by GOAT deletion (See Figure 4B). This networks summary is presented in Table 4.

**Table 4:** Regulator Effects networks, identified by Ingenuity Pathway Analysis platform.

**Regulators**	**Consistency score**	**Target genes in the dataset**	**Diseases and functions**	**Predicted relationship**	**Known regulator/disease relationship**
FSH	3	*↑Cyp11a1, ↑Cyp19a1, ↑Fshr, ↑Fst, ↑Inhba, ↑Inhbb, ↑Nppc, ↑Tk1, ↓Wt1*	Development of female reproductive tract; Development of genital organ	Activation	2/2
↓ELF4	1.155	*↓Cdkn1a, ↓Cxcl2, ↓Spp1*	Cell movement of phagocytes	Inhibition	0/1
↑STAT1	−4.536	*↓Angpt2, ↓Apoe, ↑C3, ↓Cdkn1a, ↓Cxcl2, ↓Edn1, ↓Serpina3*	Cell movement of phagocytes	Inhibition	1/1
TNF	−6.791	*↓Apoe, ↓Bmper, ↓Cdkn11a, ↑Col1a1, ↓Cyp26b1, ↓Edn1, ↑Frzb, ↑Fst, ↓Mndal, ↑Igf2, ↑Naglu, ↓Ngfr, ↓Sox9, ↑Th, ↑Thbs2, ↑Tnfrsf1b, ↑Vegfc*	Development of sensory organ	Activation	1/1
IL1A	−7.506	*↓Bcl3, ↑Il18, ↑Tac1*	Inflammation of the limb	Activation	0/1
CSF2	−8.89	*↑C3, ↓Cd74, ↓Cdkn1a, ↓Cxcl2, ↓Edn1, ↑Inhba, ↓Spp1, ↑Tnfrsf1b*	Cell movement of phagocytes	Inhibition	1/1
EPO	−11.023	*↓Angpt2, ↓Cdkn1a, ↓Edn1, ↑Il18, ↓Spp1, ↑Vegfc*	Cell movement of phagocytes	Inhibition	1/1
LIF	−11.431	*↓Cdkn1a, ↓Cxcl2, ↑Cyp19a1, ↓Serpina3, ↓Spp1, ↑Tac1*	Cell movement of phagocytes	Inhibition	0/1
IFNα	−11.839	*↑C3, ↓Cdkn11a, ↓Cxcl2, ↓Il17ra, ↓Il6r, ↑Mmp28*	Cell movement of phagocytes	Inhibition	1/1
IFNγ	−14.500	*↓Bcl3, ↑Il18, ↓Itgb2, ↑Tac1*	Inflammation of the limb	Activation	1/1

*The networks are scaled by a consistency score, a measure of how causally consistent and densely connected a network is. A red up arrow indicates an increase in the dataset; A green down arrow indicates a decrease in the dataset*.

### qRT-PCR Analysis of Key DEGs in Juvenile GOAT KO Mouse Ovaries

We next performed a qRT-PCR assessment of the top DEGs in juvenile WT and GOAT KO mice, to more specifically identify individual genes that might be influenced by the absence of GOAT. While expression of certain ovarian genes can be influenced by estrous cycle stage, cyclicity was not controlled in the study to avoid additional handling and stress associated with vaginal smearing, and the clear role for ghrelin in regulation of the stress response (106). Gene expression analysis confirmed the absence of *Mboat4* transcript in the juvenile GOAT KO mouse ovaries, in which no amplification was observed (data not shown). In the juvenile ovary, there was no significant correlation between the RNA sequencing and RT^2^ PCR array in the fold changes of the six top under-expressed genes assessed. This absence of correlation was specifically due to a different direction of change in the expression of *Myh6*, which was under-expressed in the RNA sequencing and over-expressed in the RT^2^ PCR array (Table 5). *Leprotl1*, a gene closely involved in the negative regulation of growth hormone (GH) receptor expression and intracellular protein trafficking (86), was under-expressed in both the RNA sequencing and the RT^2^ PCR array.

**Table 5:** RT^2^-PCR array gene expression in juvenile and adult GOAT KO mice.

**Gene symbol**	**Gene name NCBI**	**Juvenile ovary fold change (*p-*value)**	**Adult ovary fold change (*p-*value)**
*Grik3*	Glutamate receptor, ionotropic, kainate 3	2.7 (^*^0.01)	1.30 (0.14)
*Spocd1*	SPOC domain containing 1	2.6 (^*^0.01)	0.74 (0.36)
*Grem1*	Gremlin 1, DAN family BMP antagonist	1 (0.97)	1.67 (0.53)
*Cyp19a1*	Cytochrome P450, family 19, subfamily a, polypeptide 1	0.6 (0.07)	0.81 (0.76)
*Inhba*	Inhibin, beta A	0.8 (0.24)	0.9 (0.81)
*Hspb7*	Heat shock protein family B (small) member 7	1.1 (0.63)	2.85 (0.33)
*Sohlh1*	Spermatogenesis and oogenesis specific basic helix-loop-helix 1	1.7 (^*^0.05)	0.91 (0.66)
*Drd4*	Dopamine receptor D4	0.6 (0.14)	1.11 (0.73)
*Leprotl1*	Leptin receptor overlapping transcript-like 1	0.8 (^*^0.02)	0.73 (^*^0.04)
*Dcdc2b*	Doublecortin domain containing 2b	1 (0.90)	1.08 (0.68)
*Cited4*	Cbp/p300-interacting transactivator, with Glu/Asp-rich carboxy-terminal domain, 4	0.8 (0.45)	0.97 (0.93)
*Rab3b*	RAB3B, member RAS oncogene family	1 (0.92)	0.35 (^*^0.03)
*Rab6b*	RAB6B, member RAS oncogene family	0.9 (0.63)	2.02 (0.09)
*Myh6*	Myosin, heavy polypeptide 6, cardiac muscle, alpha	3.5 (^*^ 0.01)	0.35 (0.14)

*Gene expression changes in GOAT KO mice ovaries relative to WT controls*.

* *p < 0.05*.

There was a significant positive correlation between RNA sequencing and RT^2^ PCR array fold changes of the top over-expressed genes (*r* = 0.66, *p* = 0.04). This was driven principally by significant increases in *Grik3*, a glutamate ionotropic receptor encoding gene and an excitatory target in the ovary (66, 107), and *Spocd1*, a gene involved in negative regulation of phosphatase activity (68, 69).

### Effects of GOAT on the Mature Ovary and the Capacity to Breed

Since GOAT KO mice had a pronounced reduction in the number of ovarian follicles (per mm^3^ of ovarian tissue) as juveniles, coupled with changes in genes and gene pathways closely involved in the regulation of reproductive development and function, we next examined if these effects were likely to be carried through into adulthood and if the breeding capacity of these mice was likely to be altered.

**Figure 5:**
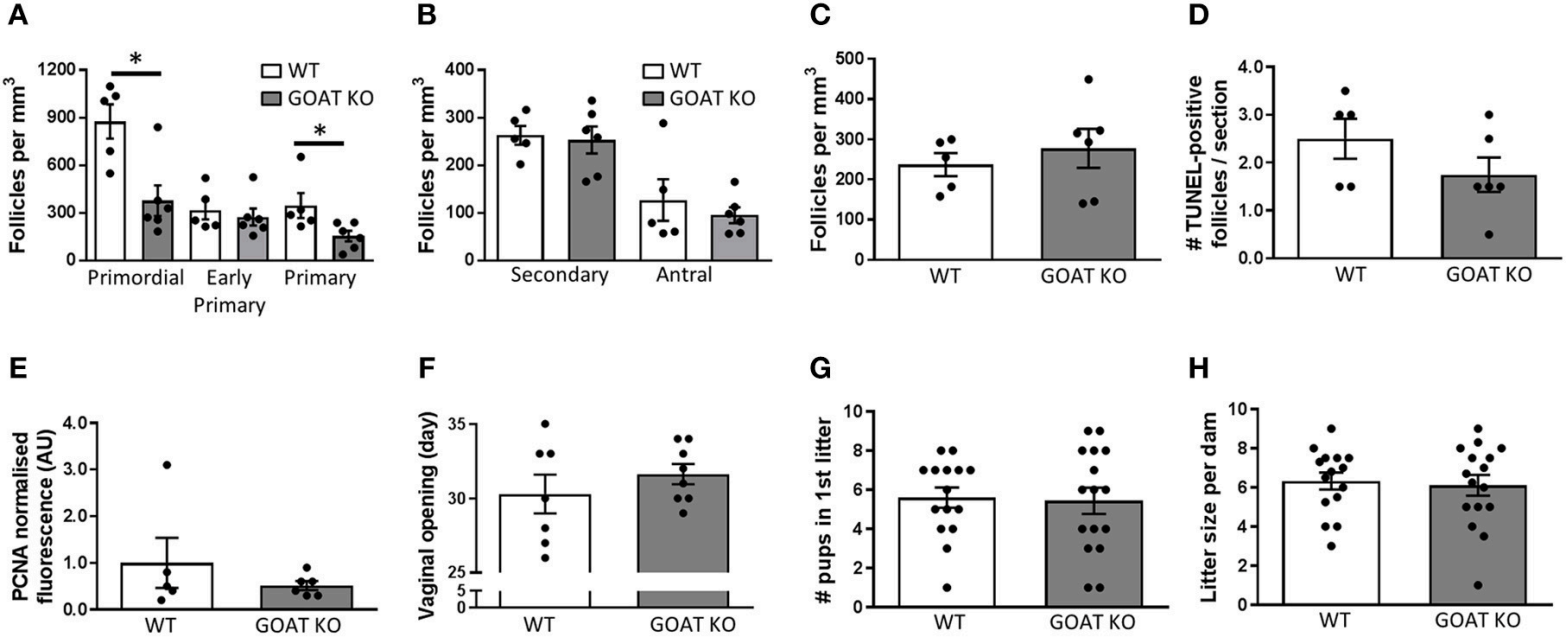
Effects of GOAT deletion on adult ovarian follicles, reproductive development and breeding capacity. **(A)** Small follicle; **(B)** Large follicle; and **(C)** Atretic follicle counts in the ovary of adult GOAT KO and WT mice. Follicle numbers are expressed per mm^3^ of ovarian tissue. **(D)** Number of TUNEL-positive follicles in the ovary of adult GOAT KO and WT mice. **(E)** Normalized mean fluorescence intensity of proliferating cell nuclear antigen (PCNA) staining in adult GOAT KO and WT ovaries. **(F)** Day of vaginal opening. **(G)** Mean number of pups born in the first litter. **(H)** Mean number of pups born in all litters to GOAT KO and WT mice. Data are expressed as mean ± SEM. *p < 0.05.

The reduction in the number of follicles seen in juvenile GOAT KO mice persisted into adulthood, with significantly reduced numbers of primordial [*t*_(_9) = 3.46, *p* = 0.007, Figure 5A; expressed per mm^3^] and primary follicles [*t*_(_9) = 2.39, *p* = 0.04, Figure 5A; expressed per mm^3^]. There were, again, no differences in the populations of large healthy and atretic follicles (Figures 5B,C; expressed per mm^3^), and no effect of GOAT deletion on apoptosis (TUNEL) or proliferation (PCNA) markers able to detect changes primarily in large follicles (Figures 5D,E). Of all the top 10 over- and under-expressed DEGs identified in GOAT KO juvenile ovaries by RNA sequencing, only two were also altered in adult GOAT KO ovaries: *Rab3b*, involved in exocytosis (93, 94), and *Leprotl1*, which was also under-expressed in the juvenile phase and is involved in the regulation of GH action (86, 87).

Despite these remaining subtle effects of GOAT deletion on the mature ovary, the age at puberty onset was not affected in these mice (Figure 5F). Encouragingly, there were also no differences between GOAT KO and WT mice in the number of pups born in the dam's first litter (Figure 5G) or in all litters (data not shown), suggesting pregnancy was not compromised. There were also no differences in the numbers of litters produced by continuously mated dams within a 3–5 month period (Figure 5H) or in the time between litters (data not shown). These data suggest that despite acyl ghrelin's regulatory role in reproductive development and pregnancy (31, 33, 34), it is not necessary for successful reproduction, at least not under institutional breeding facility conditions during the peak of the reproductive lifespan.

## Discussion

Acylated and des-acylated ghrelin peptides regulate multiple physiological functions, including reproduction [reviewed in (18)]. While the function of des-acyl ghrelin is not yet fully elucidated, and its receptor is currently unknown, there is now substantial evidence to support its independent role in a number of physiological conditions (9, 10, 24, 108). Here, we show that genetic deletion of GOAT, an enzyme responsible for the acylation of ghrelin that thus leads to an absence of acyl and a chronic increase in the levels of des-acyl ghrelin, resulted in long-term changes in ovarian morphology, as well as changes in gene pathways associated with reproductive development and function. These changes were not reflected in the reproductive maturation timeline or breeding capacity, suggesting that while GOAT KO mice do not have an overt reproductive phenotype, some of their underlying biological functionality is notably different from that in WT mice. These findings therefore have important implications for future studies employing this global knockout model, as well as for the greater understanding of ghrelin's role in reproductive development. These data may also indicate a degree of functional redundancy within the ghrelin system to ensure reproductive success, similar to a substantial functional redundancy that exists within the gonadotropin-releasing hormone (GnRH) neuronal population, where the presence of only 12% of GnRH neurons is sufficient for pulsatile gonadotropin release and puberty onset, and 12% to 34% are sufficient for the control of estrous cyclicity in the mouse (109). It may therefore be possible that while the presence of GOAT and acyl ghrelin at certain levels is essential for optimal development of the ovary, these may not be essential in maintaining fertility. It is also important to consider that in GOAT KO mice a certain compensation may occur in the context of life-long absence of acyl ghrelin. These mice have significantly elevated levels of des-acyl ghrelin, compared to WT controls (37). While the receptor for des-acyl ghrelin remains to be discovered, it is now acknowledged to have an independent bioactivity, that alternatively counteracts or mimics the actions of acyl ghrelin. As such, des-acyl ghrelin has been shown to reduce the levels of acyl ghrelin (24) and to normalize acyl-ghrelin induced changes in insulin and glucose levels (29), while it does not affect acyl ghrelin-induced GH, prolactin or adrenocorticotropic hormone production (29, 30). It does, however, mimic the inhibitory effects of acyl ghrelin on luteinising hormone (LH) release (30). It is plausible that at least to some degree the elevated des-acyl ghrelin levels in GOAT KO mice exert compensatory effects driven by the absence of acyl ghrelin, through GHSR-independent pathways.

Our characterization of the ovarian transcriptome in juvenile mice revealed that although the number of DEGs between the two genotypes represented a relatively small subset of genes, these genes were associated with several biological processes and functions regulating reproductive development. As such, *Grem1, Cyp19a1, Inhba*, and *Sohlh1*, that were among the top ten upregulated transcripts, play a critical role in folliculogenesis. *Grem1*, expressed in the granulosa cells of developing follicles (110), regulates primordial to primary follicle transition, by antagonizing the members of the bone morphogenetic protein (BMP) family (111), such as the anti-Müllerian hormone (AMH) that controls the activation of primordial follicles into the growing follicle pool (71). *Sohlh1*, that is required for oogenesis, is also expressed in postnatal ovary where it is confined to primordial oocytes (81, 112). *Sohlh1* expression, together with other oocyte-specific transcription factors, is essential for primordial follicle activation (113), with its absence leading to follicular arrest (81). *Inhba* expression is associated with follicular growth, regulating cell proliferation and FSH action in the ovary (76, 105, 114, 115). Together with an aromatase-encoding gene, *Cyp19a1*, that was also significantly upregulated in the ovaries of GOAT KOs, these transcripts modulate endocrine signaling (116).

Overall, increased expression of the above transcripts suggests the GOAT KO juvenile ovary may exhibit advanced follicle maturation, growth, and recruitment of primordial follicles into the growing pool. When we assessed the numbers of ovarian follicles (per mm^3^ of ovarian tissue), we saw a significant reduction in the presence of small follicles (primordial and early primary in juveniles, and primordial and primary follicles in adults). Secondary and antral were not affected by GOAT deletion at any age and this was further confirmed by the absence of follicular atresia, apoptosis, and proliferation in these follicle populations. These data suggest that by three weeks of age the number of primordial follicles (at least when expressed per mm^3^ of ovarian tissue) are already significantly reduced in GOAT KO mice, but these primordial follicles are not excessively recruited to grow, at least not at this age. It remains to be established whether the reduction in the number of primordial follicles in these mice is driven by a reduction in the number of embryonic germ cells; by excessive apoptosis during the mitotic-meiotic transition [embryonic days (E) 13.5–15.5]; or during the nest breakdown and primordial follicle pool formation (E17.5-P5), typically associated with a significant wave of germ cell loss and oocyte death [reviewed in (117)]; or whether this occurs later during postnatal development. It would be also of interest to examine, in future studies, if any alterations in gonadal development are also evident in male GOAT KO mice. Nevertheless, the decline in the small follicle populations in GOAT KO mice (per mm^3^ of ovarian tissue), particularly primordial follicles, is possibly indicative of an accelerated exhaustion of the ovarian reserve and a shortened reproductive lifespan (118). While this also remains to be explored in future studies, the reduction in the number of the primordial follicles was not associated with changes in reproductive development and function. We found no differences in the onset of puberty in GOAT KOs, as well as no changes to the reproductive capacity of these mice, as also noted in initial studies using this global knockout model (37, 40). It is important to note, however, that our assessments were conducted under standard non-stressed laboratory housing conditions and that the mice were not assessed into the period of expected reproductive senescence. We have previously shown that GOAT KO mice are more anxious than WT animals, under stressed conditions (10). GOAT is also essential for survival in a calorie-restricted environment (37). It therefore remains to be established whether the reproductive capacity of GOAT KOs is affected in a suboptimal environment, and how the increased depletion of the primordial follicles affects the timing of cessation of the reproductive lifespan.

In addition to differences in ovary specific genes and processes in juvenile GOAT KO mice, we observed significant changes in genes contributing to cell signaling and immune pathways, as identified by the pathway enrichment analysis, using the Reactome and KEGG databases, as well as by the IPA platform. For instance, two top enriched canonical pathways included *EIF2 Signaling* and *Complement System*. eIF2 (eukaryotic initiation factor-2) initiates protein translation and synthesis in ribosomes. Phosphorylation of eIF2 is among the first steps in response to cellular stress and apoptosis (119), and the *EIF2 Signaling* pathway is significantly enriched in human primordial oocytes during the transition from primordial to primary stage (120). The complement system integrates the interaction between the innate and adaptive immune responses, and its major role is the clearance of immune complexes and apoptotic cells (121). An upregulation of the *Complement System* pathway in xenobiotic-treated neonatal mouse ovaries has been suggested to underlie xenobiotic-induced ovotoxicity and primordial follicle apoptosis (122), and may be associated with the reduction in the numbers of small follicles in the GOAT KOs in our study.

Our use of the IPA platform, in addition to the Reactome and KEGG databases of pathway enrichment analysis, allowed us to make predictions for what potential upstream regulators may be modulating the DEGs in the juvenile GOAT KO ovaries, and what downstream biological functions they affect. In this analysis we focused on downstream functions associated with inflammatory diseases and disorders, as well as functions associated with organismal development, as we identified these to be most reflective of the changes in our dataset. As the result of this analysis, FSH was predicted as the main upstream regulator, affecting the expression of several genes in the dataset (*Wt1, Tk1, Cyp11a1, Fst, Cyp19a1, Fshr, Nppc, Inhba, Inhbb*), subsequently driving the *development of genital organ* and the *development of female reproductive tract*, the top biological functions associated with *Reproductive System Development and Function*, as identified by the IPA platform in our dataset. The overall predicted activation state of these downstream biological functions is once again suggestive of premature ovarian development in the GOAT KOs, which may be the cause of the significant reduction in the number of primordial follicles (per mm^3^) in these mice. The number of primordial follicles is a major predictor of the female reproductive lifespan. In the mammalian ovary, the vast majority of germ cells are lost before the primordial follicle formation. In the mouse ovary, the establishment of primordial follicle pool is completed by P7 (123). By P19, approximately half of these follicles are already depleted and in the post-pubertal ovary at P45, only a third of the initial population of primordial follicles are left (124). After this phenomenal loss, only a small proportion of primordial follicles will be recruited into the growing follicle pool and reach ovulation, while the remainder of the primordial follicle pool continues to gradually decline during the period of sexual maturity, until only ~4% of the primordial follicle population is left at 12 months of age (124). The period of pubertal development therefore represents an important milestone of primordial follicle depletion. This extensive depletion appears to be gonadotropin driven, and while the exact mechanisms are unknown, pubertal increases in the levels of FSH and LH that drive folliculogenesis are also likely to indirectly drive primordial follicle depletion, since GnRH antagonism during the peri-pubertal period prevents the significant primordial follicle loss that typically occurs during this time (103, 125, 126). Our sequencing data from the juvenile ovary did not reveal changes in the expression of BCL-2 modifying factor (BMF), which has recently been identified as a critical promoter of fetal oocyte and prepubertal primordial follicle loss (103, 127). However, differential expression of genes driving reproductive development and their potential regulation by FSH, as indicated by our upstream regulator analysis, warrant investigation of the quantity and quality of the follicle pool in the fetal and early postnatal GOAT KO ovary in future studies. Importantly, mutations in several genes that had significant contribution to the pathway analyses in our study, such as inhibin genes, including *INHBA*, as well as *SOHLH1* and *FSHR* are associated with premature ovarian failure in humans, and these genes are among the potential candidate genes responsible for this condition (128–130).

In summary, here, for the first time, we have characterized the ovarian gene and follicle profiles, as well as the reproductive potential of female GOAT KO mice, a model that through a genetic deletion of GOAT results in an absence of circulating acyl and high levels of des-acyl ghrelin (37). Our findings indicate that while the ovarian transcriptome and follicles in these animals are affected by the global deletion of GOAT, their reproductive capacity is unchanged. Although global gene knockout may induce widespread developmental effects, these data suggest that while a presence of acyl ghrelin supports ovarian development, as is the case in WT mice, its absence is not detrimental for successful reproduction. Our data also suggest that substantial reduction in ovarian follicle numbers (per mm^3^ of ovarian tissue) can be sustained without overt detrimental effects on the ability to reproduce at least not during the peak of the reproductive capacity.

## Author Contributions

LS, ZA, and SS conceived of and designed the work. LS, JG, ZA, and SS (i.e., all authors) made substantial contributions to the acquisition, analysis and interpretation of data. LS and SS wrote the manuscript. All authors critically revised it for important intellectual content.

### Conflict of Interest Statement

The authors declare that the research was conducted in the absence of any commercial or financial relationships that could be construed as a potential conflict of interest.
