# Supplementary material for: Acylated Ghrelin Supports the Ovarian Transcriptome and Follicles in the Mouse: Implications for Fertility
Source: Front Endocrinol (Lausanne). 2019 Jan 15;9:815. doi: 10.3389/fendo.2018.00815 (PMC6340924; doi:10.3389/fendo.2018.00815)
Supplement: Supplementary file 1 [file Table_1.pdf]

sTable 1. Top canonical pathways that were significantly affected by GOAT deletion as identified by Ingenuity® Pathway Analysis (IPA). The significance of the association between upregulated genes and the canonical pathway was evaluated using a right-tailed Fisher's exact test to calculate a p-value determining the probability that the association is explained by chance alone. Ratios referring to the proportion of upregulated genes from a pathway related to the total number of molecules that make up that particular pathway are also displayed.

Treatment	Ingenuity Canonical Pathways	Genes	-log(p-value)	Ratio
GOAT KO	Virus Entry via Endocytic Pathways	CD55,RRAS2,PIK3C2A,PIK3R1,ITGA6,ITGB6,PRKD3,CXADR,PRKCZ,ACTA1,PRKCB	5.32	0.129
	NF-κB Activation by Viruses	RELA,CCR5,RRAS2,PIK3C2A,PIK3R1,ITGA6,AKT3,PRKD3,PRKCZ,PRKCB	5.11	0.132
	Role of Pattern Recognition Recepto	PTX3,TLR4,RELA,PIK3C2A,PIK3R1,MAPK8,C1QB,PRKD3,PRKCZ,PRKCB	4.43	0.111
	LPS-stimulated MAPK Signaling	TLR4,RELA,RRAS2,PIK3C2A,PIK3R1,MAPK8,PRKD3,PRKCZ,PRKCB	4.28	0.117
	HER-2 Signaling in Breast Cancer	RRAS2,PIK3C2A,PIK3R1,PARD6B,AKT3,ITGB6,PRKD3,PRKCZ,PRKCB	4.28	0.118
	MSP-RON Signaling Pathway	TLR4,CCL2,PIK3C2A,PIK3R1,MST1R,PRKCZ,ACTA1	4.23	0.163
	Endothelin-1 Signaling	PIK3C2A,ITPR2,PIK3R1,PTGS1,MAPK8,PLA2G2A,PRKCZ,RRAS2,EDN1,CASQ1,PLD6,PRKD3,PRKCB	4.05	0.080
